# Expanding the Molecular Spectrum of *HK1*-Related Charcot-Marie-Tooth Disease, Type 4G; the First Report in Iran

**DOI:** 10.34172/aim.2023.43

**Published:** 2023-05-01

**Authors:** Masoumeh Goleyjani Moghadam, Zohreh Elahi, Mohamad Soveyzi, Sanaz Arzhangi, Shahriar Nafissi, Hossein Najmabadi, Kimia Kahrizi, Zohreh Fattahi

**Affiliations:** ^1^Genetics Research Center, University of Social Welfare and Rehabilitation Sciences, Tehran, Iran; ^2^Kariminejad - Najmabadi Pathology & Genetics Center, Tehran, Iran; ^3^Iranian Neuromuscular Research Center (INMRC), Tehran University of Medical Sciences, Tehran, Iran; ^4^Department of Neurology, Shariati Hospital, Tehran University of Medical Sciences, Tehran, Iran

**Keywords:** Charcot-Marie-Tooth type 4G, Whole exome sequencing, *HK1* gene

## Abstract

Charcot-Marie-Tooth disease type 4G (CMT4G) was first reported in Balkan Gypsies as a myelinopathy starting with progressive distal lower limb weakness, followed by upper limb involvement and prominent distal sensory impairment later in the patient’s life. So far, CMT4G has been only reported in European Roma communities with two founder homozygous variants; g.9712G>C and g.11027G>A, located in the 5’-UTR of the *HK1* gene. Here, we present the first Iranian CMT4G patient manifesting progressive distal lower limb weakness from 11 years of age and diagnosed with chronic demyelinating sensorimotor polyneuropathy. Whole-exome sequencing for this patient revealed a homozygous c.19C>T (p. Arg7*) variant in the *HK1* gene. This report expands the mutational spectrum of the *HK1*-related CMT disorder and provides supporting evidence for the observation of CMT4G outside the Roma population. Interestingly, the same Arg7* variant is recently observed in another unrelated Pakistani CMT patient, proposing a possible prevalence of this variant in the Middle Eastern populations.

## Introduction

 Hereditary motor and sensory neuropathy (HMSN), also known as Charcot-Marie-Tooth (CMT), is the most common type of peripheral neuropathy, with a prevalence of 1 in 2500 worldwide.^1^ The disease has high clinical and genetic heterogeneity, leading to different subtypes.^2^ Autosomal recessive forms of CMT (ARCMT) account for less than 10% of the patients in Europe but are more frequent (30%–50%) in populations with high rates of consanguineous marriages, such as populations in the Mediterranean region, Middle East and also the Roma/Gypsy populations in Europe.^3^

 Charcot-Marie-Tooth disease type 4G (CMT4G), also named hereditary motor and sensory neuropathy-Russe (HMSNR) type (OMIM #605285), is a form of ARCMT, characterized by the age of onset in the first decade, distal muscle weakness progressing to severe in lower limbs, delayed early motor development, prominent distal sensory impairment and mildly reduced motor nerve conduction velocities (demyelinating range).^4,5^

 In 2009, the molecular basis of HMSNR was elucidated and refined to the *HK1* gene. So far, only two homozygous variants in the 5’ untranslated region of the *HK1* gene have been reported to cause HMSNR. The first variant is c.-290G > C (NM_001358263.1), formerly known as g.9712G > C (NM_033498), located in an alternative untranslated exon (AltT2), which was then described as a founder mutation in the HMSN-Russe type in the Spanish gypsy population. The second variant; c.-196 + 1241G > A (NM_001358263.1), formerly known as g.11027G > A (NM_033498), is located in complete linkage disequilibrium with g.9712G > C variant. However, based on the nucleotide location and conservation, the g.9712G > C variant is favored to be the pathogenic mutation. To our knowledge, the above-mentioned homozygous variants in the *HK1* gene are the only mutations reported for HMSNR phenotype and only detected in patients of Roma/Gypsies from Bulgaria, Spain, Slovakia, and the Czech Republic.^4-7^ Today, mutations in this gene are considered one of the common causes of ARCMT in European Roma communities.^6^

 In 2021, Kanwal et al reported five consanguineous CMT families from Pakistan, in which one of the patients carried the homozygous c.19C > T (p. Arg7*) in the *HK1* gene (NM_001358263.1). The patient was an 11-year-old boy with demyelinating CMT, who showed symptoms from one year of age, presenting with muscle atrophy, sensory loss, and foot deformities. This patient was the first and only report of CMT4G caused by a mutation in *HK1* other than founder mutations in the Roma/Gypsy population.^8^

 Given the high prevalence of consanguineous marriages in Iran, ARCMT is expected to be highly prevalent in the Iranian population, and different types of demyelinating or axonal ARCMTs are reported in Iranian patients, such as CMT4A (*GDAP1* gene), CMT4B1 (*MTMR2* gene), CMT4C (*SH3TC2* gene), CMT4D (*NDRG1* gene), CMT4F (*PRX* gene), CMT4H (*FGD4* gene), CMT1F (*NEFL* gene), CMT2A2B (*MFN2* gene), CMT2S (*IGHMBP2* gene), CMT2T (*MME* gene).^9-13^

 Here, we present an Iranian 21-year-old female manifesting progressive muscle weakness in distal limbs from 11 years of age who has been diagnosed with chronic demyelinating sensorimotor polyneuropathy. Interestingly, the same homozygous variant described by Kanwal et al,^8^ c.19C > T (p. Arg7*) in the *HK1* gene, was also detected in this patient. To our knowledge, this is the first observation of CMT4G in the Iranian population. We expand the molecular spectrum of the *HK1*-related CMT disorder and provide supporting evidence for the observation of CMT4G outside the Roma population.

## Case Report

**Figure 1:**
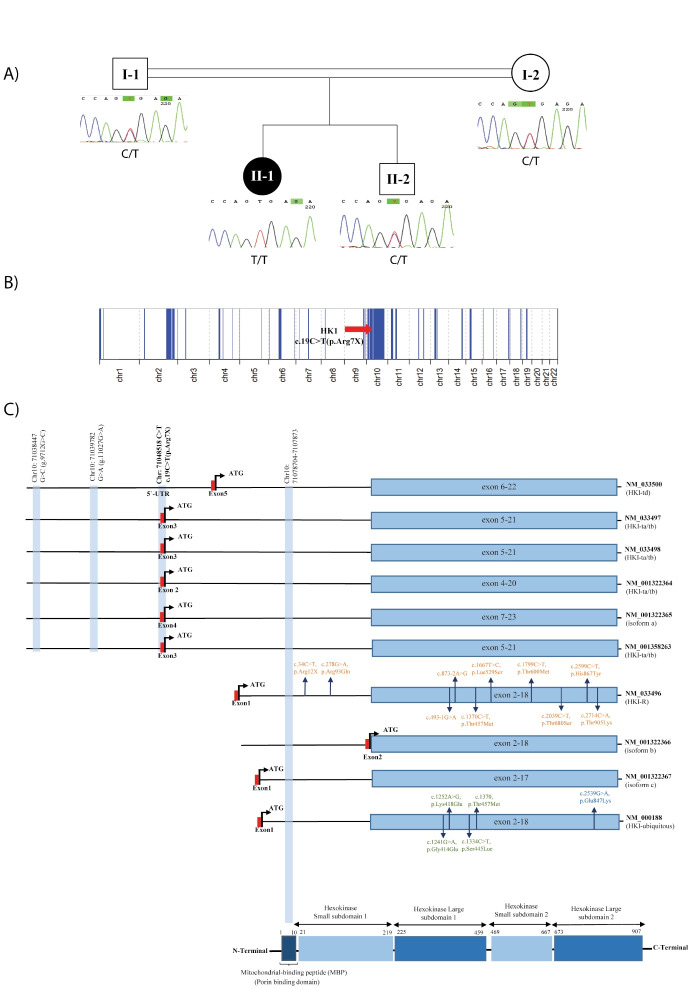
Schematic representation of the genetic results obtained in this study. **(A)** Patient's pedigree, Sanger sequencing results show that the c.19C > T (p. Arg7*) variant in the *HK1* gene co-segregates with the phenotype; **(B)** Schematic representation of the detected ROH regions in this patient; **(C)** Schematic representation of the *HK1* protein domains and transcripts, showing the location of the variant in this study (p.Arg7*) compared to the previously reported variants in CMT4G phenotype (g.9712G > C, g.11027G > A). Also, the location of reported variants in other phenotypes are shown in orange for hemolytic anemia due to hexokinase deficiency (NM_033496.2), green for neurodevelopmental disorder with visual defects and brain anomalies and blue for retinitis pigmentosa 79 (NM_000188)

 A 14-year-old girl was referred to the Genetics Research Center with walking difficulty and unstable gait. Her healthy parents were consanguineous (first cousins), and she had a healthy male sibling (Figure 1A). Her psychomotor development was normal and her muscle weakness started at the age of 11 years. She had numbness in lower limbs and there was no history of hearing or visual impairment. Her mother had normal term delivery with an unremarkable pregnancy and her motor development was normal. She had no speech delay or cognitive impairment. Neurological examination at the age of 14 revealed normal mental status, normal language and memory, weakness of intrinsic hand muscles and severe lower distal weakness, causing bilateral foot drop. She had muscle atrophy in her upper and lower extremities, which was less pronounced in the upper limb. She had an unstable gait and was not able to walk on her toes or heels.

 She also had reduced deep tendon reflexes. There was also slightly reduced sensation in the lower limbs. The electrophysiological study (nerve conduction velocity) showed chronic demyelinating sensorimotor polyneuropathy with uniform conduction slowing. Motor conduction velocity in the upper extremities was 21-27 m/s but was unobtainable in the lower extremities. Subsequently, CMT1A was excluded by the initial screening of the *PMP22* gene duplication using the MLPA method.

 Whole-exome sequencing was performed to identify the disease-causing variant in this family. After obtaining informed consent according to the ethical committee’s recommendations, the proband’s DNA was isolated from peripheral blood, used for exome enrichment by the Agilent SureSelect Human All Exon V5 (Agilent Technologies Inc., Santa Clara, CA, USA) enrichment kit, and sequenced by the HiSeq Illumina sequencer machine (Illumina, San Diego, CA, USA). The generated Fastq file was aligned to hg19 assembly (GRCh37) of the human reference genome with Burrows-Wheeler Aligner (0.7.17-r1188)^14^ and then further processed using the GATK pipeline (v.4.1.4.1), adhering to the best practices.^15^ The mean depth of coverage for the exons of the human genome based on CCDS Release 22 was 94.47X, with 95.8% and 92.8% coverage at 10x and 20x, respectively. The final rare candidate variant detected through the standard filtering pipeline was eventually validated by Sanger sequencing, and co-segregation study was performed using the proband’s, her healthy parents’, and her healthy sibling’s DNA. After performing the standard pipeline of WES analysis, copy number variations (CNVs) and runs of homozygosity (ROH) intervals were detected by GATK germline CNV caller (v.4.1.4.1) and AutoMap algorithm (v1.2),^16^ respectively.

 Whole-exome sequencing in the proband led to the identification of a homozygous likely pathogenic c.19C > T (p. Arg7*) variant in the *HK1* gene (NM_001358263.1), which co-segregates in the family (Figure 1A). This variant is absent in public genomic databases, while no homozygous loss of function variants along the whole *HK1* gene are seen in population databases. It creates a premature translational stop signal (p. Arg7*) in the first coding region of most *HK1* isoforms and is predicted to cause loss of normal protein function either through protein truncation or nonsense-mediated mRNA decay (Table 1).

**Table 1:** Description of the Causative Variant in This Patient

**Gene**	**Transcript**	**Variant**	**Exon**	**CADD PHRED Score**	**Other Bioinformatics Predictions**	**Frequency in gnomAD **	**Frequency in Iranome **	**ROH Interval**	**ACMG Classification**
*HK1 *	Hexokinase-1 isoform HKI-ta/tb (NM_001358263.1) MANE Plus Clinical isoform	chr10:71048518 C > T; c.19C > T (p.Arg7*)	3 (First coding exon)	35	Mostly damaging	NA	NA	chr10:51633027- 81270173 (29.64 Mb)	Likely pathogenic
	Hexokinase-1 isoform a (NM_01322365.1)		4 (First coding exon)						
	Hexokinase-1 isoform HKI-ta/tb (NM_033498.2) Founder mutations reported isoform		3 (First coding exon)						
	Hexokinase-1 isoform HKI-ta/tb (NM_033497.2)		3 (First coding exon)						
	Hexokinase-1 isoform HKI-ta/tb (NM_001322364.1)		2 (First coding exon)						
	Hexokinase-1 isoform HKI-td (NM_033500.2)	chr10:71048518 C > T; c.-123C > T	5'-UTR (exon3) (Non-coding)						
	Hexokinase-1 isoform HKI (NM_000188.3) MANE Select Canonical isoform	Not located	Not located						
	Hexokinase-1 isoform c (NM_001322367.1)								
	Hexokinase-1 isoform b (NM_001322366.1)								
	Hexokinase-1 isoform HKI-R (NM_033496.2)								

 Regarding the consanguineous background of this patient, 280.02 Mb of her chromosomes were in homozygous regions, in which the c.19C > T (p. Arg7*) variant is located in a long 29.64 Mb ROH interval (chr10:51633027-81270173), supporting the causality of this variant in the patient’s phenotype (Figure 1B).^17^ As shown in Figure 1C, the detected variant in this study is located in the critical HMSN-Russe region present in specific *HK1* isoforms involved in the CMT4G phenotype, similar to the previously-known g.9712G > C founder mutation in the Roma population. Therefore, we speculate that this variant may lead to comparable consequences underlying the CMT4G phenotype in this patient.

## Discussion

 In this study, we reported an Iranian patient presenting the *HK1*-related CMT phenotype due to a stop-gain homozygous variant in this gene. The present case is the first observation of CMT4G in the Iranian population. It is also the second report of CMT4G phenotype outside European Roma communities. As described above, this patient is clinically similar to the previous CMT4G patients of Roma communities. She also shares a similar phenotype with the Pakistani patient with the same mutation described by Kanwal et al.^8^

 The* HK1* gene encodes hexokinase 1 protein, which is involved in the phosphorylation of glucose to glucose-6-phosphate in the glycolytic pathway to produce energy. The C-terminal half of the enzyme is conserved among the different isoforms and contains the catalytic site of hexokinase, and the N-terminal half is thought to pertain to regulatory functions. There is also a 5-prime porin binding domain (PDB), also known as mitochondrial-binding peptide (MBP), which is responsible for the protein binding to the mitochondria (Figure 1C). The HK1 protein is ubiquitously expressed and is also called a brain-type hexokinase. It is significantly expressed in tissues highly dependent on glucose utilization, such as the brain, erythrocytes, testis, etc.^18,19^

 The 5´ untranslated exons of the *HK1* gene, located in the critical HMSN-Russe region, undergo extensive alternative splicing generating different isoforms, some of which are tissue-specific, i.e. *HK-1* (ubiquitous, NM_000188), *HK-R* (erythrocytes, NM_033496), *HK-TA* (testis-A, NM_033497), *HK-TB* (testis-B, NM_033498), *HK-TD* (testis-D, NM_033500), and etc.^20,21^

 These isoforms correspond to a range of different phenotypes related to this gene. Biallelic variants in the catalytic domains of *HK-R* isoform (NM_033496) are associated with hemolytic anemia due to hexokinase deficiency (OMIM #235700), while the two homozygous variants in the 5’-UTR of the gene (NM_033498) are known as the cause of neuropathy, hereditary motor and sensory, Russe type (OMIM #605285). Moreover, a rare heterozygous missense variant, c.2539G > A (p.E847K), in the main ubiquitous somatic isoform (NM_000188) of *HK1* has been reported to cause retinitis pigmentosa 79 with an autosomal dominant pattern of inheritance (OMIM #617460).^22^ Recently, four *de novo* variants located in the conserved catalytic domain shared among all the isoforms are reported to be involved in the pathogenesis of a neurodevelopmental disorder with visual defects and brain anomalies (OMIM# 618547, Figure 1C).^6,19,23^

 In 2000, Andreoni et al characterized six additional exons (T1-T6 exons) in the 5’ region of the specific *HK1* mRNAs in spermatogenic cells. These isoforms are the product of alternative splicing of different exons in this region, in which the PBD is absent in all, producing a non-bound HK1 protein to the mitochondria.^24^ Later in 2009, Hantke et al could detect the g.9712G > C variant responsible for HMSNR. The causal variant was located in an alternative untranslated exon (AltT2) in the same 5’ region sequences (between T1 and T2 exons) not present in the somatic HKI isoform. These testis-specific AltT2-containing transcripts were also detected in neural tissues (although with lower expression compared to the ubiquitous somatic isoform) and with different expression patterns between newborn and adult peripheral nerves and brain tissues. The highest expression of *HK1* was detected in myelinated axons compared to unmyelinated axons and Schwann cells (SCs). However, investigating the functional consequences of the g.9712G > C variant revealed no difference in the *HK1* expression pattern (using an antibody against the PBD). They also reported no evidence of hexokinase catalytic activity in cultured SCs from HMSNR patients (Figure 1C).^6^

 Although the pathogenic mechanism of these variants in HMSNR is unknown, Hantke et al proposed that these AltT2-containing transcripts may be involved in developmental regulation based on the differential expression pattern between newborn and adult peripheral nerves. They also speculated the disruption in *HK1* translation as the possible mutational mechanism in HMSNR.

 The c.19C > T (p. Arg7*) variant detected in the patient of this study is also located in the 5’ region and also the first coding exons of these specific transcripts (Table 1), whereas it is not located in the ubiquitous canonical and somatic HK1 isoform (NM_000188). Therefore, we speculate that the p. Arg7* variant is responsible for the CMT4G phenotype in our patient by a similar mutational mechanism affecting the *HK1* translation as it causes a premature stop codon in these specific isoforms.

 In conclusion, we expand the molecular spectrum of the *HK1*-related CMT disorder and provide another evidence for the observation of CMT4G outside the European Roma population. The observation of the same Arg7* variant in another unrelated CMT patient from the Middle East proposes a possible prevalence of this variant in the region, which requires the report of additional patients to be proved.

## References

[1] Kitani-Morii F, Noto YI (2020). Recent advances in Drosophila models of Charcot-Marie-Tooth disease. Int J Mol Sci.

[2] Jani-Acsadi A, Krajewski K, Shy ME (2008). Charcot-Marie-Tooth neuropathies: diagnosis and management. Semin Neurol.

[3] Dubourg O, Azzedine H, Verny C, Durosier G, Birouk N, Gouider R (2006). Autosomal-recessive forms of demyelinating Charcot-Marie-Tooth disease. Neuromolecular Med.

[4] Sevilla T, Martínez-Rubio D, Márquez C, Paradas C, Colomer J, Jaijo T (2013). Genetics of the Charcot-Marie-Tooth disease in the Spanish Gypsy population: the hereditary motor and sensory neuropathy-Russe in depth. Clin Genet.

[5] Šafka Brožková D, Haberlová J, Mazanec R, Laštůvková J, Seeman P (2016). HSMNR belongs to the most frequent types of hereditary neuropathy in the Czech Republic and is twice more frequent than HMSNL. Clin Genet.

[6] Hantke J, Chandler D, King R, Wanders RJ, Angelicheva D, Tournev I (2009). A mutation in an alternative untranslated exon of hexokinase 1 associated with hereditary motor and sensory neuropathy -- Russe (HMSNR). Eur J Hum Genet.

[7] Gabrikova D, Mistrik M, Bernasovska J, Bozikova A, Behulova R, Tothova I (2013). Founder mutations in NDRG1 and HK1 genes are common causes of inherited neuropathies among Roma/Gypsies in Slovakia. J Appl Genet.

[8] Kanwal S, Choi YJ, Lim SO, Choi HJ, Park JH, Nuzhat R (2021). Novel homozygous mutations in Pakistani families with Charcot-Marie-Tooth disease. BMC Med Genomics.

[9] Mohammadi Pargoo E, Aryani O, Tonekaboni SH, Kamalidehghan B, Houshmand M (2012). A novel mutation of GDAP1 associated with Charcot-Marie-Tooth disease in an Iranian family. Iran J Child Neurol.

[10] Georgiou DM, Nicolaou P, Chitayat D, Koutsou P, Babul-Hirji R, Vajsar J (2006). A novel GDAP1 mutation 439delA is associated with autosomal recessive CMT disease. Can J Neurol Sci.

[11] Taghizadeh S, Vazehan R, Beheshtian M, Sadeghinia F, Fattahi Z, Mohseni M (2020). Molecular diagnosis of hereditary neuropathies by whole exome sequencing and expanding the phenotype spectrum. Arch Iran Med.

[12] Wang H, Kaçar Bayram A, Sprute R, Ozdemir O, Cooper E, Pergande M (2019). Genotype-phenotype correlations in Charcot-Marie-Tooth disease due to MTMR2 mutations and implications in membrane trafficking. Front Neurosci.

[13] Jamiri Z, Khosravi R, Heidari MM, Kiani E, Gharechahi J (2022). A nonsense mutation in MME gene associates with autosomal recessive late-onset Charcot-Marie-Tooth disease. Mol Genet Genomic Med.

[14] Li H, Durbin R (2009). Fast and accurate short read alignment with Burrows-Wheeler transform. Bioinformatics.

[15] Van der Auwera GA, Carneiro MO, Hartl C, Poplin R, Del Angel G, Levy-Moonshine A, et al. From FastQ data to high confidence variant calls: the Genome Analysis Toolkit best practices pipeline. Curr Protoc Bioinformatics 2013;43(1110):11.10.1-11.10.33. 10.1002/0471250953.bi1110s43. PMC4243306 25431634

[16] Quinodoz M, Peter VG, Bedoni N, Royer Bertrand B, Cisarova K, Salmaninejad A (2021). AutoMap is a high performance homozygosity mapping tool using next-generation sequencing data. Nat Commun.

[17] Wakeling MN, Laver TW, Wright CF, De Franco E, Stals KL, Patch AM (2019). Homozygosity mapping provides supporting evidence of pathogenicity in recessive Mendelian disease. Genet Med.

[18] Griffin LD, Gelb BD, Wheeler DA, Davison D, Adams V, McCabe ER (1991). Mammalian hexokinase 1: evolutionary conservation and structure to function analysis. Genomics.

[19] Okur V, Cho MT, van Wijk R, van Oirschot B, Picker J, Coury SA (2019). De novo variants in HK1 associated with neurodevelopmental abnormalities and visual impairment. Eur J Hum Genet.

[20] Jamwal M, Aggarwal A, Palodi A, Sharma P, Bansal D, Maitra A (2019). A nonsense variant in the Hexokinase 1 gene (HK1) causing severe non-spherocytic haemolytic anaemia: genetic analysis exemplifies ambiguity due to multiple Isoforms. Br J Haematol.

[21] Travis AJ, Foster JA, Rosenbaum NA, Visconti PE, Gerton GL, Kopf GS (1998). Targeting of a germ cell-specific type 1 hexokinase lacking a porin-binding domain to the mitochondria as well as to the head and fibrous sheath of murine spermatozoa. Mol Biol Cell.

[22] Wang F, Wang Y, Zhang B, Zhao L, Lyubasyuk V, Wang K (2014). A missense mutation in HK1 leads to autosomal dominant retinitis pigmentosa. Invest Ophthalmol Vis Sci.

[23] van Wijk R, Rijksen G, Huizinga EG, Nieuwenhuis HK, van Solinge WW (2003). HK Utrecht: missense mutation in the active site of human hexokinase associated with hexokinase deficiency and severe nonspherocytic hemolytic anemia. Blood.

[24] Andreoni F, Ruzzo A, Magnani M (2000). Structure of the 5’ region of the human hexokinase type I (HKI) gene and identification of an additional testis-specific HKI mRNA. Biochim Biophys Acta.

